# Supplementary material for: FastqPuri: high-performance preprocessing of RNA-seq data
Source: BMC Bioinformatics. 2019 May 3;20:226. doi: 10.1186/s12859-019-2799-0 (PMC6500068; doi:10.1186/s12859-019-2799-0)
Supplement: Supplementary file 2 — Archive of FastqPuri. Archive containing all files needed to install and run FastqPuri v1.0.6. Date stamp March 22, 2019. (GZ 47,819 kb) [file 12859_2019_2799_MOESM2_ESM.gz › FastqPuri-1.0.6/html/globals_defs.html]

FastqPuri: File Members


FastqPuri


### - \_ -

- \_\_isascii\_c
  : str\_manip.c

### - a -

- ADAP
  : defines.h
- ADAP2
  : defines.h
- ALL
  : defines.h

### - b -

- B\_LEN
  : defines.h
- BASESPERCHAR
  : defines.h
- BITSPERCHAR
  : defines.h
- BLOOM
  : defines.h
- bool
  : defines.h

### - c -

- CONT
  : defines.h
- CONT2
  : defines.h

### - d -

- DEFAULT\_MINL
  : defines.h
- DEFAULT\_MINQ
  : defines.h
- DEFAULT\_NQ
  : defines.h
- DEFAULT\_NTILES
  : defines.h

### - e -

- ENDS
  : defines.h
- ENDSFRAC
  : defines.h
- ERROR
  : defines.h

### - f -

- FA\_ENTRY\_BUF
  : defines.h
- false
  : defines.h
- FALSE\_POS\_RATE
  : defines.h
- FRAC
  : defines.h

### - g -

- GLOBAL
  : defines.h
- GOOD
  : defines.h
- GOOD2
  : defines.h

### - k -

- KMER\_LEN
  : defines.h

### - l -

- LOG\_4
  : defines.h
- LOWQ
  : defines.h
- LOWQ2
  : defines.h

### - m -

- max
  : defines.h
- MAX\_FASZ\_TREE
  : defines.h
- MAX\_FILENAME
  : defines.h
- MAX\_RCOMMAND
  : defines.h
- mem\_usage
  : defines.h
- mem\_usageMB
  : defines.h
- min
  : defines.h
- MIN\_NMATCHES
  : defines.h

### - n -

- N\_ACGT
  : defines.h
- NFILES\_DS
  : defines.h
- NFILTERS
  : defines.h
- NNNN
  : defines.h
- NNNN2
  : defines.h
- NO
  : defines.h
- NPOOL\_1D
  : defines.h
- NPOOL\_2D
  : defines.h

### - s -

- STRIP
  : defines.h

### - t -

- T\_ACGT
  : defines.h
- TREE
  : defines.h
- TRIM\_STRING
  : trim.c
- true
  : defines.h

### - z -

- ZERO\_POS\_RATE
  : defines.h
- ZEROQ
  : defines.h


---

Generated on Mon Mar 19 2018 23:42:01 for FastqPuri by  

 1.8.14
